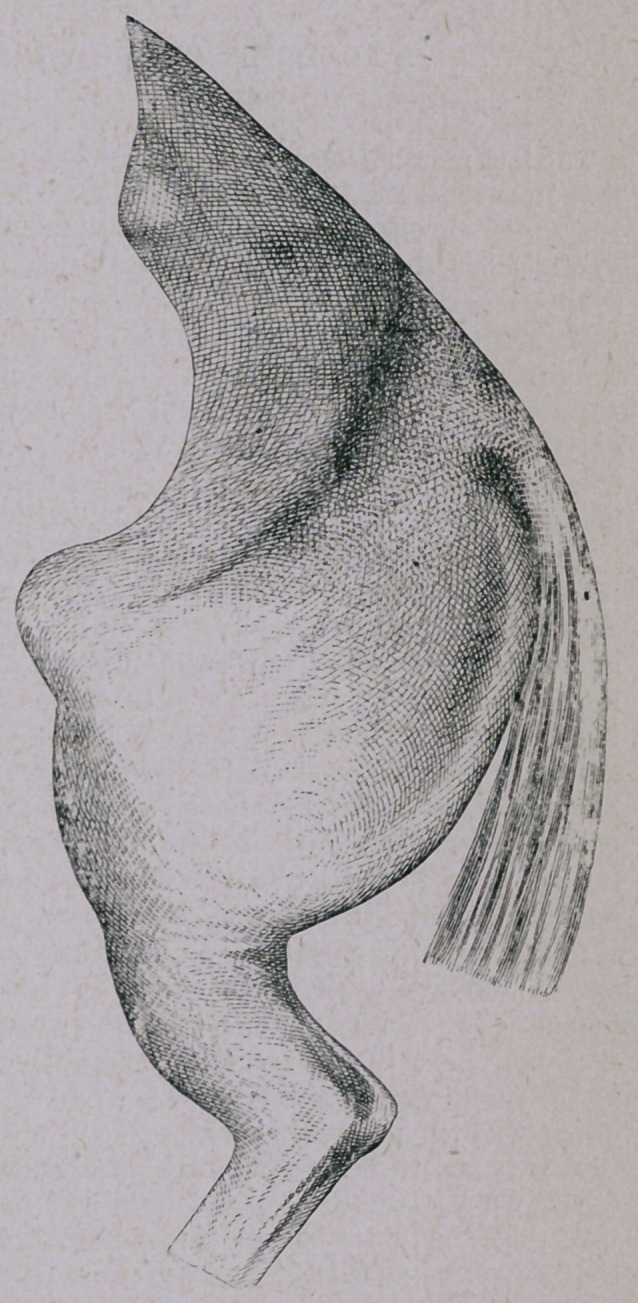# Correspondence

**Published:** 1885-07

**Authors:** Thomas Waller

**Affiliations:** Royal Veterinary College, Edinburgh


### CORRESPONDENCE.

ROYAL VETERINARY COLLEGE,
EDINBURGH, May 29, 1885.

*To the Editors of the* JOURNAL OF COMPARATIVE MEDICINE AND SURGERY:

GENTLEMEN—In perusing the various articles contained in your very excellent April number, there are two that have particularly attracted my attention.

The first is the paper on "Tuberculosis," by my friend Professor Bang, a copy of which, in the Danish language, he was good enough to forward to me; but not being acquainted with the Danish tongue, I was reluctantly compelled to lay it aside—and it is a paper possessing a special interest to me, for the simple reason that I have taken up a rather definite position in connection with the subject of milk contamination by tuberculous cows. While I cannot agree with all the conclusions arrived at by Prof. Bang, I must, nevertheless, bear my individual testimony to the great interest and value possessed by his paper and the experiments he therein records. The position I have personally taken up in connection with milk contamination is, "that there can be no contamination of milk by the tubercle virus without local (mammary) lesions." If the virus of this disease was conveyed from point to point by the blood stream, instead of, as we know to be the case, by the lymph stream, one could understand that the milk could become virulent in a tuberculosis localized in the lungs, liver, or any other organ; not only so, the saliva, the urine and other secretions would become virulent also. In making this remark, I do not, of course, lose sight of the fact that we occasionally meet with cases of *general infective tuberculosis;* but these are comparatively rare, only result from the involvement of blood-vessels (as in the lungs), and when existent produce such an effect upon the system as, practically to arrest the function of such secerning gland as the udder.

The conclusions arrived at by Prof Bang are largely dependent upon the assumption that he is enabled in all cases to diagnose positively the existence during life of tubercular mammitis. I confess that I do not pretend to be able to make such diagnoses with accuracy. I have met with cases of induration of the udder, which presented no characteristic differing from those of tubercular induration, and in which the animal has not shown the slightest indication of being affected with tuberculosis, either in her general condition or in her parental history, and in which a cure has been readily established by the adoption of appropriate therapeutical measures; at the same time I am free to acknowledge that cases occasionally come under observation in which, by the aid of collateral evidence, *e. g.*, the animal's general condition, her previous history, the existence of nymphomania or enlarged lymphatic glands, one is enabled to give a positive opinion anent the character of the mammary lesion.

Since writing the "Four Bovine Scourges," to which Prof. Bang alludes, I have met with a greater proportion of cases of tubercular mastitis than I had seen previous to the date of its publication, and some of these cases have occurred in Danish cows; but in every instance the udder lesions *have been*

*secondary*, and I cannot conceive the possibility of a local, primary contamination of the mammary gland from any source other than direct or indirect inoculation, seeing that each lacteal orifice is guarded by a powerful sphincter.

I am quite prepared to grant that the results of properly conducted experiments are more conclusive than are theories, no matter how good may be the basis upon which the latter are founded; but, notwithstanding this acknowledgment, I cannot divest my mind of the idea that, in the cases of successful infection by milk from animals in whose udders there were apparently no tubercular lesions, there must have been local, mammary lesions which had escaped observation. Whichever view may ultimately be found to be the correct one, on one point all who are interested in the question of the relation of the infective diseases of animals to those of man will agree with me when I say that the paper, published in your JOURNAL, by Prof. Bang, possesses the very highest merit, and should be of the greatest possible value to those who are engaged in the study of this important subject.

The second paper which has interested me is that by Mr. Thos. B. Rogers, on so-called "Azoturia" in the horse not only from the fact of his dealing with the affection mentioned, but from his having, incidentally, drawn attention to an atrophic muscular condition of the kind by which he looks upon as one of its sequelæ.

I am very glad to find that not only on your side of the Atlantic, but in Germany also, the subject just mentioned has attracted attention, and that the opinion expressed by Mr. Rogers, as also that expressed by Bollinger, in your translation of an article by him which appeared in your October number, coincide to a very large extent with the views held and taught by myself in this school for some years—certainly for the past seven or eight. Two years ago, in a paper read by me at a meeting of the North of Ireland V. M. Association, held at Belfast, I incidentally touched upon what I have long thought was a great mistake, viz., the use of the word "Azoturia" in designating this affection, my objection to the word being that it gave no indication as to the pathology of the affection, but merely referred to a characteristic symptom of it. In my earliest lectures on the subject I suggested the use of the word "Azotæmia," but subsequently I taught, and do now teach, that the disease is to all intents and purposes a form of "Acute Uræmia," and that, in all probability, the organ seemingly at fault is the liver. In an article in the *Veterinary Journal* subsequently to the appearance of my remarks at Belfast, and in answer to a criticism thereon, I explained more fully my views as to the nature of the affection.

An attempt has been made to identify the disease with albuminaria, but I may remark, in passing, that neither my colleague, Dr. Aitken, or myself, have been able satisfactorily to demonstrate the presence of albumen in the urine in anything like sufficient quantities to bring about the conditions which exist in this malady; and even if it were proved that albuminaria is an invariable phenomenon, I should not myself attach much importance to it, seeing that we have ample evidence of the fact that there is extensive disintegration of the albumenoids, in the immense quantity of urea always present in the urine. That there is also, as is so much insisted upon by Bollinger, more or less destruction of the red cells and liberation of hæmatin, must be patent to all, as from no other source than this can we get the coloring matter in the urine, unless, indeed, it were derived from the muscles—a supposition, I may remark, that cannot be entertained, seeing that the circumstances under which the disease makes its appearance are not at all favorable to the disintegration of muscular tissue, in fact, rather the contrary.

That a hæmolytic process goes on there can be no doubt, but there must, necessarily, exist considerable doubt as to its seat or origin, and there must exist the same doubt as to its being sufficient to produce the phenomena associated with and characteristic of the affection under consideration.

If hæmo-globinomia is sufficient in itself to produce these phenomena, why do we not get identical manifestations in Red-water of Cattle? Granted that in some districts this affection is associated with neural and cerebral disturbance, such disturbance is in thousands of cases never manifested at all, and yet there can be no question as to the presence of enormous quantities of both albumen and hæmatin in the urine. Again, there is no brain disturbance seen in the disease known as "Sanguimears Ascites" (Simonds) in the sheep, except such as would naturally follow the anæmia, which is necessarily a marked feature in the malady as it is in Red-water.

In hæmoglobin-uria, produced by the injection of strong ammonia into the circulation, I have seen no evidence of cerebral disturbance after the passing off of the primary convulsions; neither does such exist in the hæmoglobin-uria, sometimes seen in animals which have been exposed to the effects of the gases liberated (especially carbon menoxide) in the process of combustion in the accidental burning of stables.

I cannot for one moment endorse the theory, held by some, that Azoturia is associated with, and is due to, nephritis—neither in the clinical characters of the disease or in the condition of the kidneys after death have I seen any evidence of it, any more, for that matter, than there is in Red-water.

That the malady is a blood disorder there can, I think, be no reasonable doubt, and that the liver is the organ largely concerned, directly or indirectly, in its production, there can be as little doubt; but as to what may be the precise nature of the forces engaged in its production, there is very much doubt. I have thought it might be due to the accumulation in the blood of some of those chemical agents of whose nature we know so little, *e. g.*, tyrosin, leucho, etc., or to uric or hippuric compounds; and most certainly I entertain the opinion, and for over twenty years have done so, that Red-water is due to the presence of excessive quantities of alkaline phosphates in the blood, as the hæmoglobin-uria of horses after exposure to fire is due to the action of one or more hæmolytic gases with which the blood becomes changed by pulmonary inhalation.

In reference to the treatment recommended by Mr. Rogers, I confess I should hesitate to give the large doses of morphia mentioned by him, nor can I understand its action, except on the supposition that it exerts an antidotal effect to the poisonous principles accumulated in the blood. I have always recommended morphia injections, or the use of belladonna and chloroform for the purpose of modifying and controlling the convulsions (when present) of acute uræmia, but I have trusted mainly to the action of powerful eliminatives and respiratory and cardiac stimulants; and, in addition, have attached great importance to the washing out of the bladder with alkaline solutions.

In connection with a sequel of acute uræmia, mentioned by Mr. Rogers, viz., "Atrophy of the Crural Muscles," I may observe that I have never met with it, and if I did I should look upon it as being due to injury inflicted upon the muscles and the crural nerve by the violent convulsive movements so often associated with the disease, or by strain received during the attempts of the patient to maintain the standing position or to regain its feet when prostrate. But, while I have not met with this lesion as sequela of acute uræmia, it is familiar to me as a result of severe injury inflicted upon the crural muscles and nerves.

Some years ago I reported several of the secases in the *Veterinary Journal*, and the sketch I send herewith was reproduced therein to show the condition of the muscles and the great prominence into which the patella is thrown by their absorption. The sketch was roughly drawn by myself from the living subject, some thirteen years ago, and elaborated subsequently by Mr. Henry Ashbie, M.R.C.V.S.—then a student in this College—and in this particular case the stifle had been severely blistered by a practitioner who looked upon the case as one of laxation of the patella. The first case of the kind which came under my notice occurred in the practice of Mr. Lawson, of Manchester, some fifteen years ago, and in that case the lesion was produced by a severe blow from the buffer of a locomotive; my diagnosis being based simply on the peculiar action of the limb in the first place, and the extensive subsequent atrophy of the muscles in the second.

An almost identical lesion is sometimes seen in the Caput muscles of the fore-limb, as the result of severe injury. I had, some years ago, four cases in Iceland ponies under my charge, at one and the same time, the injury being produced by the rough usage to which the animals had been subjected during a stormy voyage from Reijkavig to Leith; and in each instance the prominent symptom was a counterpart of that seen in Crural strain, viz., inability to support the weight of the body when it was thrown on the injured limb.

I am, yours faithfully,

THOMAS WALLER.

## Figures and Tables

**Figure f1:**